# Meta-analysis of high-intensity interval training effects on cognitive function in older adults and cognitively impaired patients

**DOI:** 10.3389/fphys.2025.1543217

**Published:** 2025-03-06

**Authors:** Wenting Zhang, Shuyi Zeng, Yao Nie, Keke Xu, Qiyuan Zhang, Yu Qiu, Yongqiang Li

**Affiliations:** ^1^ School of Sport and Health Sciences, Nanjing Sport Institute, Nanjing, Jiangsu, China; ^2^ Rehabilitation Medicine Center, Jiangsu Zhongshan Geriatric Rehabilitation Hospital, Nanjing, Jiangsu, China; ^3^ Rehabilitation Medicine College, Nanjing Medical University, Nanjing, Jiangsu, China; ^4^ Rehabilitation Medicine Center, The First Affiliated Hospital of Nanjing Medical University, Nanjing, Jiangsu, China

**Keywords:** high-intensity interval training, cognitive function, moderateintensity continuous training, exercise, cognitive flexibility, attention

## Abstract

**Background:**

Cognitive enhancement treatments are limited, and while High-Intensity Interval Training (HIIT) has been suggested to improve cognitive function, high-quality evidence remains scarce. This meta-analysis evaluates the effects of HIIT on cognitive performance compared to moderate-intensity continuous training (MICT) and control groups in older adults and cognitively Impaired Patients.

**Methods:**

A systematic search of PubMed, Embase, and Cochrane Library databases was conducted for articles published until 10 October 2024. Eighteen studies were included, comparing cognitive outcomes across HIIT, MICT, and control groups. Cognitive tests evaluated included the Stroop test, Digit Span Test (DST), Trail Making Test (TMT), and the MOST test.

**Results:**

HIIT significantly improved performance compared to MICT in the Stroop test (SMD = −0.8, 95% CI: −1.3 to −0.2) and DST (SMD = 0.3, 95% CI: −0.0–0.5). Compared to control groups, HIIT significantly enhanced performance in the TMT (SMD = −0.7, 95% CI: −1.3 to 0.0) and MOST test (SMD = −1.2, 95% CI: −1.8 to −0.7).

**Conclusion:**

This meta-analysis supports the efficacy of HIIT in enhancing cognitive functions, particularly in cognitive flexibility, working memory, task switching, attention control, and inhibitory control. These findings suggest that HIIT can be an effective intervention for improving cognitive behavior in older adults and cognitively Impaired Patients.

**Systematic Review Registration:**

https://www.crd.york.ac.uk/PROSPERO/, Identifier CRD42023413879.

## 1 Introduction

Over the past few decades, a great number of studies have revealed numerous benefits of exercise for physical health (Fiuza-Luces et al., 2018; D’Onofrio et al., 2023; Sellami et al., 2021), particularly its positive impact on the brain and cognitive function (Stillman et al., 2020; Mandolesi et al., 2018; Hertzog et al., 2008). Cognitive function refers to the ability of the brain to process information, solve problems, and perform tasks, including executive functions, memory, language skills, and cognitive flexibility. As the aging population continues to grow, the importance of maintaining and enhancing cognitive function becomes increasingly prominent. Therefore, investigating how exercise positively affects cognitive function has become a critical area of study.

High-intensity interval training (HIIT) has garnered considerable attention as a time-efficient and effective exercise method. This training modality involves alternating periods of high-intensity exercise with rest or low-intensity exercise for recovery (Wewege et al., 2018). Previous applications and studies have primarily focused on training methods for athletes, and HIIT has been shown to significantly enhance their performance in sports (Reindell and Roskamm, 1959; Reindell, 1962; Billat, 2001). Not until the 21st century, did the research focus shift to the clinical applications of HIIT, particularly its potential for cognitive function improvement and brain rehabilitation (Calverley et al., 2020; Poon et al., 2023; LaCount et al., 2022). Several studies have already confirmed that HIIT offers greater health benefits compared to traditional moderate-intensity continuous training (MICT) (Stensvold et al., 2020; Cuddy et al., 2019; Taylor et al., 2020). The physiological mechanisms underlying these effects may include increased Brain-Derived Neurotrophic Factor (BDNF), which could all support cognitive function (Jiménez-Maldonado et al., 2018).

Despite the growing body of research on HIIT and cognitive function, current findings remain inconsistent. Some studies have found positive effects of HIIT on cognitive function. For instance, the study by Gjellesvik et al. (2021) suggests that HIIT can improve executive functions; Drollette and Meadows (2022) propose improvements in working memory; Tian et al. (2021) report enhanced cognitive flexibility. However, other studies failed to observe significant effects, as stated by Sokołowski et al. (2021), who found no association between HIIT and cognitive performance. Nicolas Hugues’ (Hugues et al., 2021) article reviews the therapeutic application of HIIT in stroke rehabilitation. However, most of the article focuses on changes in serum biomarkers related to the treatment, without clearly describing the specific behavioral rehabilitation outcomes. These studies and reviews primarily target elderly individuals or populations after a stroke, both of which are characterized by cognitive deficits. Therefore, this paper aims to synthesize and categorize these populations to investigate the effects of HIIT on cognitive reshaping.

This meta-analysis aims to provide a comprehensive assessment of HIIT’s effects on cognitive function, clarify its physiological mechanisms, and offer practical guidance for implementing HIIT in cognitive rehabilitation, especially for populations at risk of cognitive decline.

## 2 Methods

This systematic review was designed in accordance with the Preferred Reporting Items for Systematic Reviews and Meta-Analyses (PRISMA) guidelines (Liberati et al., 2009) and followed the Cochrane systematic review guidelines for literature search and selection (Moher et al., 2009).

### 2.1 Search strategy

Computerized searches were conducted in PubMed, Embase, and Cochrane Library databases for articles published until 10 October 2024. Boolean search methods were employed with the following mesh terms: [“high-intensity intermittent exercise” OR “high-intensity intermittent training”] AND [“cognitive accessibility” OR “cognitive balance”]. The search strategy is detailed in Appendix). All articles considered in the search were restricted to peer-reviewed publications written in English.

### 2.2 Selection criteria

To determine the inclusion criteria for the study, we followed the PICOS framework. Population: The study population included different age groups, such as children, adolescents, adults, and elderly individuals. It encompassed both healthy individuals and those with physical and mental illnesses, athletes and trained individuals, All participants exhibited some degree of cognitive and awareness decline, but their physical health status was sufficient to perform exercise modalities such as HIIT and MICT. The search was not restricted by age, race, or gender, which can provide a more comprehensive understanding of the effects of the intervention across different demographic groups. Intervention: High-intensity exercise was defined as exercise intervals lasting up to 5 min, which elicited a peak heart rate of at least 80%. These intervals were characterized by rest or light exercise. Any form of exercise that met these intensity criteria (e.g., treadmill running, cycling, whole-body exercises) was included. There were no limitations on the duration of HIIT interventions. Both acute HIIT interventions and long-term HIIT interventions lasting several months were included in the review. Comparison: The controls were those who did no exercise and continued their regular daily activities or were engaged in reading. Additionally, the comparison between HIIT and MICT was included in the search. MICT is defined as exercise lasting no less than 20 min and performed at a maximum heart rate of 75% or lower, without short rest or lighter exercise periods. Outcome: Cognitive function assessments including the Stroop Test, Digit Span Test (DST), TMT and The More-odd Shifting Task (MOST). Study design: the predominant study design was randomized controlled trial (RCT).

After the article search by an initial reviewer, two reviewers screened the study titles and abstracts for further selection. Any discrepancies were resolved through discussion with a third reviewer until a consensus was reached.

### 2.3 Quality assessment

**FIGURE 1:**
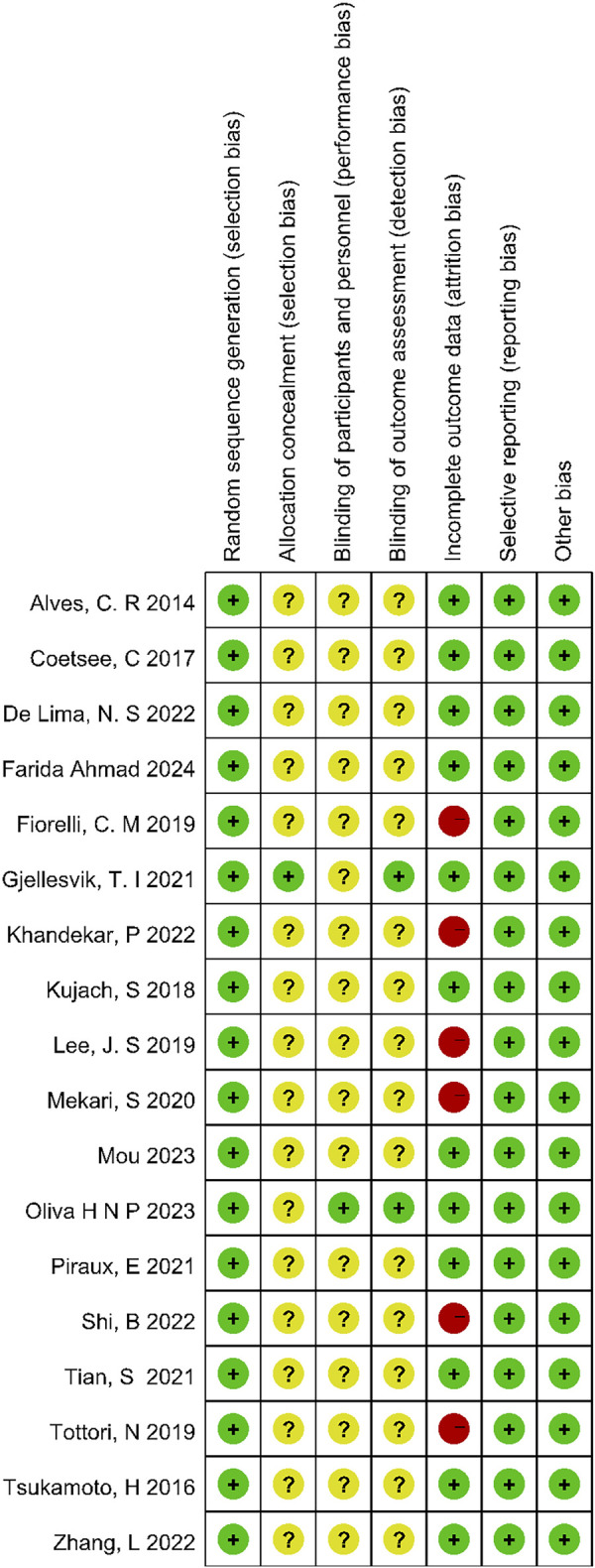
Methodological quality of included studies.

**FIGURE 2:**
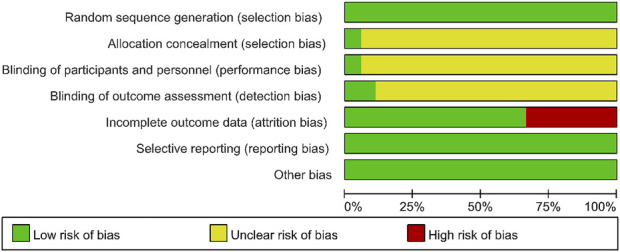
The distribution of the methodological quality of included studies.

The risk of bias within each domain of the included studies was assessed using a domain-based assessment tool from the Cochrane Collaboration (Higgins et al., 2019) (Figure 1), Since most of the included studies employed a crossover design, allocation concealment and blinding were not reported, indicating potential methodological weaknesses. Figure 2 presents the proportion of methodological quality items across the studies. The quality assessment was conducted by two reviewers, with any discrepancies resolved by consulting a third reviewer.

### 2.4 Data extraction

Titles and abstracts of all identified studies from the search were independently reviewed by two reviewers. Each reviewer created a relevant abstract list for reading and determined eligibility according to the inclusion criteria. Any discrepancies were resolved by a third reviewer. Data were extracted by one reviewer from the selected papers, which were then validated by a second reviewer. The data included for analysis encompassed the number of studies included, study design, participant numbers (including participants in the control group), details of HIIT interventions, outcome measures related to HIIT (such as Stroop, TMT, DST, MOST), and corresponding effect size.

In this study, meta-analysis was performed using a random-effects model, with the pooled effect size represented as standardized mean difference (SMD) with 95% confidence interval (95%CI). To investigate the impact of HIIT on changes in cognitive function, an overall analysis was conducted. Furthermore, sensitivity analyses were conducted to detect if specific studies contributed significantly to heterogeneity (I^2^). Based on I^2^ values, heterogeneity was evaluated as not important (0%–40%), moderate (30%–60%), substantial (50%–90%), or considerable (75%–100%) (Higgins et al., 2003). Publication bias was not evaluated due to the limited number of studies involved (less than 10). All analyses were performed using the R statistical software (version 4.2.1). Results were considered statistically significant when the p-value of the overall effect (z-value) was less than 0.05.

## 3 Results

### 3.1 Search results

**FIGURE 3:**
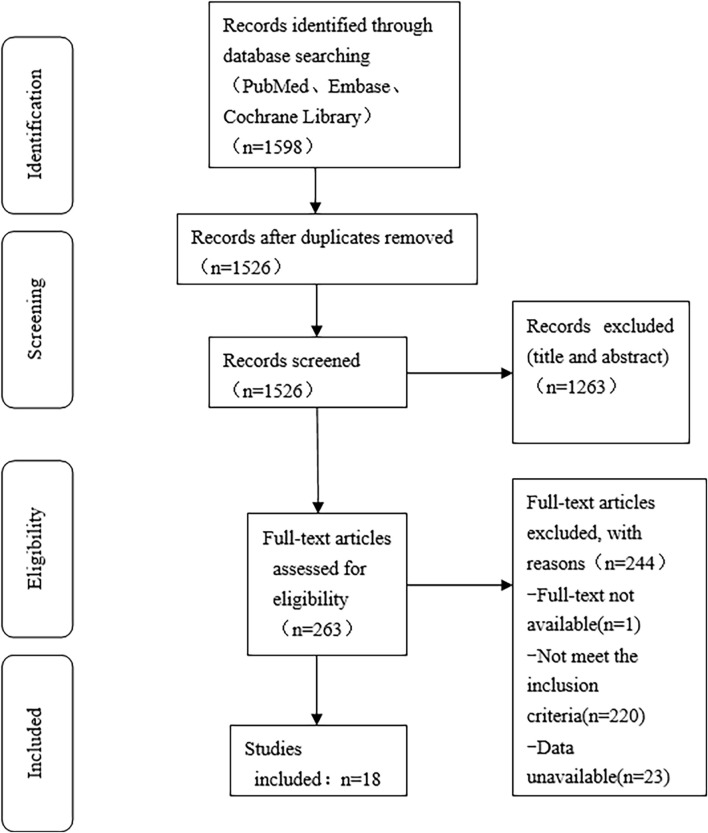
The PRISMA search flowchart.

According to the inclusion criteria, a total of 1,598 articles were retrieved from databases (PubMed, Embase, and Cochrane Library). After removing duplicate articles, 1,526 articles remained for further screening of titles and abstracts. Subsequently, 263 articles were assessed for eligibility by reading the full texts. Finally, 18 articles (Gjellesvik et al., 2021; Tian et al., 2021; Tsukamoto et al., 2016; Kujach et al., 2018; Khandekar et al., 2023; Shi et al., 2022; Alves et al., 2014; Coetsee and Terblanche, 2017; de Lima et al., 2022; Fiorelli et al., 2019; Lee et al., 2019; Mekari et al., 2020; Piraux et al., 2021; Tottori et al., 2019; Zhang et al., 2022a; Ahmad et al., 2024; Mou et al., 2023; Oliva et al., 2023) were included in the systematic review (Figure 3).

All included articles focused on the effects of HIIT on cognition. The characteristics of the included studies are provided in Table 1. In this meta-analysis, the predominant study design was RCTs, supplemented by randomized balanced crossover experiments, crossover design experiments, and randomized crossover experiments. Cognitive function was assessed primarily using cognitive scales including the Stroop Test, DST, TMT, MOST. These scales covered various cognitive domains, including executive function, attention, short-term memory, and cognitive flexibility.

**TABLE 1:** Characteristics of included studies.

Authors, country, study design	Sample	Groups(n)	Exercise intervention	Frequency (week)	Duration (weeks)	Assessments	Outcome
Alves et al. (2014) Brazil RCT (crossover design)	Healthy volunteers: n = 22, 9 males and 13 females, mean age 53.7 ± 4.7 years	Each participant received two regimens	HIIT group: warm-up for 3 min on a cycle ergometer at 60% of heart rate reserve, followed by 10 sets of 1-min high-intensity exercise at 80% of heart rate reserve, with 1-min low-intensity intervals at 60% of heart rate reserve between sets, finally a 2-min cool-down exercise at 60% of heart rate reserve Control group: a 10-min instructional session, followed by a 15-min low-intensity stretching exercise	Single session intervention	—	Stroop Test, DST	HIIIT: cognitive function improved, especially attentional control
Coetsee and Terblanche (2017) South Africa RCT	Sedentary individuals n = 67, 21 males and 46 females, mean age 62.7 ± 5.7 years	HIIT group: 13 participants MICT group: 13 participants Control group: 19 participants	HIIT group: 4 sets of 4-min running at 90%–95% of their maximum heart rate, with 3 min of active recovery at 70% of their maximum heart rate between each set MICT group: continuous walking at 70%–75% of their maximum heart rate for 47 min. Control group: no exercise	3	16	Stroop Test	HIIT group: improved information processing speed MICT group: enhanced executive function
de Lima et al. (2022) Brazil RCT	Sedentary individuals n = 25, 25 males, mean age 30–50 years old	HIIT group: 13 participants MICT group: 12 participants	HIIT group: multiple rounds of 10 sets of 20-meter sprints at 85%–100% of maximum speed, with 1 min of passive recovery between each sprint MICT group: continuous running at 60%–75% of their maximum speed for 3,500–5,000 m	3	8	DSFT, DSBT, TMT	Both groups showed improvement in cognitive function with no significant differences between the groups
Fiorelli et al. (2019) Brazil RCT (crossover design)	Parkinson’s disease patients n = 12, 6 males and 6 females, mean age 66.5 ± 8 years	Each participant underwent three different regimens	HIIT group: cycling exercises with a 4-min warm-up at a perceived load level of 9–11, followed by 7 sets of 1-min high-intensity training at a perceived load level of 15–17, interspersed with 2 min of moderate-intensity training at a load level of 9–11 MICT: cycling exercises with a 4-min warm-up at a perceived load level of 9–11, followed by 26 min of moderate-intensity training at a load level of 11–13 Control group: rest in a seated position for 30 min	Single session intervention	—	DST, TMT, Wechsler Adult Intelligence Scale-III—Associated Verbal Pairs, Symbol Search	HIIT group: improved immediate auditory memory, attention and sustained attention MICT group: improved immediate auditory memory Control group: no effect on cognition
Gjellesvik et al. (2021) Norway RCT	Patients after first stroke n = 70, 41 males and 29 females, mean age 34–72 years	HIIT group = 36 participants Control group = 34 participants	HIIT: treadmill running with a 10-min warm-up, followed by 4 sets of 4-min high-intensity exercise at 85%–95% peak heart rate, with walking intervals at 50%–70% peak heart rate Control group: no exercise	3	8	TMT-B, MoCA	HIIT group: Improved executive function
Khandekar et al. (2023) India RCT	Healthy adults n = 49, 17 males and 32 females, mean age 18–30 years	HIIT group = 26 participants Control group = 23 participants	HIIT group: cycling exercise with 4 sets of 4-min rides at 90%–95% maximum heart rate, with 3-min rides at 70% maximum heart rate as recovery intervals Control group: seat and relax	Single session intervention	—	Stroop Test, TMT	HIIT group: improved executive function
Kujach et al. (2018) Japan RCT (randomized balance design)	Sedentary adults n = 25, 16 males and 9 females, mean age 21.0 ± 1.6 years	Each participant received two regimens	HIIT group: cycle ergometer exercise with 8 bouts of 30-s intervals at 60% maximum aerobic power and 100 RPM, with 30-s rest intervals Control group: no exercise	Single session intervention	—	Stroop Test	HIIT group: improved executive function
Lee et al. (2019) Canada RCT (randomized balance design)	Children with mental disorders n = 28, 8 males and 20 females, mean age 15.5 ± 0.92 years	Each participant received two regimens	HIIT group: whole-body exercise with 3 sets of 30-s intervals above 80% maximum heart rate, with 30-s rest intervals Control group: no exercise	Single session intervention	—	The Colour-Word Stroop Task (CWST)	HIIT improved reaction efficiency and inhibitory control
Mekari et al. (2020) Canada RCT	Healthy adults n = 25, 7 males and 18 females, mean age 32 ± 8 years	HIIT group = 12 participants MICT group = 13 participants	HIIT group: cycling exercise with a 5-min warm-up, 15-s cycling at 100% output power followed by 15-s rest intervals, 2 sets of 20-min high-intensity intervals, and a final 5-min cool-down MICT group: cycling exercise with a 5-min warm-up, 34 min of cycling at 60% peak output power, and a final 5-min cool-down	3	6	Stroop Test, TMT	HIIT showed greater improvements in executive function compared to MICT
Piraux et al. (2021) Belgium RCT	Prostate cancer patients n = 72, mean age 69.1 ± 8.2 years	HIIT group = 24 participants Control group = 24 participants	HIIT group: cycling exercise with a 5-min warm-up at 65%–70% maximum heart rate, followed by 8 sets of 60-s intervals above 85% maximum heart rate, with 60-s rest intervals at a speed of 50–60 revolutions per minute Control group: no exercise	3	5–8	TMT	No significant change in cognitive function
Shi et al. (2022) China RCT (crossover design)	Healthy adults n = 66, 32 males and 34 females, mean age 19.47 ± 0.94 years	High-level exercise group: 33 participants Low-level exercise group: 33 participants	HIIT group: treadmill running with 10 sets of 1-min high-intensity exercise at 90% heart rate reserve (HRR), with 1-min intervals at 50% HRR MICT group: Treadmill running at an intensity of 40%–59% HRR for 20 min Control group: no exercise	Single session intervention	—	TMST	Both groups showed improvements in cognitive flexibility
Tian et al. (2021) China RCT (within-subject repeated measures design)	Healthy adults n = 56, 31 males and 25 females, mean age 20.18 ± 1.19 years	Each participant underwent three different regimens	HIIT group: treadmill running with 10 sets of 1-min high-intensity exercise at 90% HRR, with 1-min intervals at 50% HRR MICT group: treadmill running at 40%–59% HRR for 20 min Control group: no exercise	Single session intervention	—	TMST	Both groups showed improvements in cognitive flexibility
Tottori et al. (2019) Japan RCT	Children, n = 56, 31 males and 25 females, mean age 8–12 years	HIIT group = 27 participants Control group = 29 participants	HIIT group: aerobic and core exercises with 30-s intervals above 85% maximum heart rate, followed by 30-s rest intervals, repeated for 8–10 min. Control group: no training intervention	3	4	DSFT, DSBT, ToH	HIIT improved working memory and executive function
Tsukamoto et al. (2016) Japan RCT (randomized balance design)	Population: Healthy males n = 12, all males, mean age 22.9 ± 0.4 years	Each participant received two regimens	HIIT group: cycling exercise with 4 sets of 4-min high-intensity intervals at 90% peak VO_2_, with 3 min of low-intensity cycling at 60% peak VO_2_ as rest intervals MICT group: continuous cycling at 60% peak VO_2_ for 40 min.	Single session intervention	—	CWST	Both groups showed improvements in executive function, with HIIT demonstrating better results
Zhang et al. (2022a) China RCT	Overweight and obese children n = 72, 57 males and 15 females, mean age 11.56 ± 1.03 years	HIIT group = 24 participants Control group = 24 participants	HIIT group: treadmill running with 8 sets of 2-min high-intensity intervals at 85%–95% maximum heart rate, with 1-min relaxation intervals Control group: no exercise	Single session intervention	—	Stroop Test	HIIT improved cognitive control and inhibitory abilities
Ahmad et al. (2024) Pakistan within-group design	Healthy adults n = 34, mean age 21 ± 2 years	Each participant received two regimens	HIIT group: Participants alternate between 1 min of high-intensity exercise and 1 min of low-intensity exercise, lasting a total of 15 min MICT group: Participants engage in 20 min of moderate-intensity exercise on a treadmill	Single session intervention	—	DST	HIIT improved selective attention
Mou et al. (2023) China RCT (crossover design)	Healthy adults n = 110, 60 males and 50 females, mean age 20.17 ± 1.15 years	Each participant underwent three different regimens	HIIT group: treadmill running with 10 sets of 1-min high-intensity exercise at 90% HRR, interspersed with 1-min intervals at 50% HRR, for a total duration of 20 min MICT group: treadmill running at 40%–59% HRR for 20 min Control group: no exercise	Single session intervention	—	MOST	HIIT and MICT can both improve cognitive flexibility
Oliva et al. (2023) United States RCT (crossover design)	Healthy adults n = 26, 13 males and 13 females, mean age 24 ± 3 years	Each participant underwent three different regimens	HIIT group: a 14-min session including a 2-min warm-up, 5 intervals of 60 s at 80% HRR, interspersed with 60 s of active recovery at 30% HRR, and a 2-min cool-down MICT group: a 14-min session including a 2-min warm-up, 10 min of exercise at 60% of Heart Rate Reserve (HRR), and a 2-min cool-down Control group: no exercise	Single session intervention	—	DST	HIIT can improve executive function/semantic fluency

### 3.2 Primary outcome measures

This meta-analysis focused on the impact of HIIT on executive function. Executive function, as a fundamental aspect of cognitive process (Nguyen et al., 2019), encompasses crucial domains such as working memory, inhibitory control, and cognitive flexibility. We utilized the Stroop Test as primary outcome to quantify the effect of HIIT on executive function.

**FIGURE 4:**
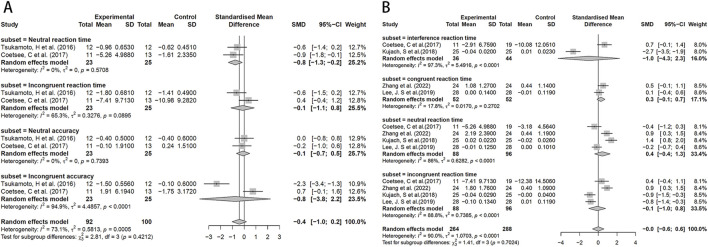
Forest plot for between-group effects of HIIT on the stroop test. **(A)** Comparison of HIIT and MICT in Stroop test results; **(B)** Comparison of HIIT and control in Stroop test.

Since the Stroop test results were expressed in multiple ways, for analysis purposes, we only selected data expressed in the same way. The analysis results showed that compared to MICT, HIIT significantly reduced neural response time (SMD = −0.8, 95%CI: −1.3 to −0.2). However, its effects on incongruent response time, neutral accuracy, and incongruent accuracy were not statistically significant (Figure 4A). No significant difference was observed in interference response time, neutral response time, and incongruent response time between the HIIT group and the control group, suggesting similar effects across studies (Figure 4B).

### 3.3 Secondary outcome measures

**FIGURE 5:**
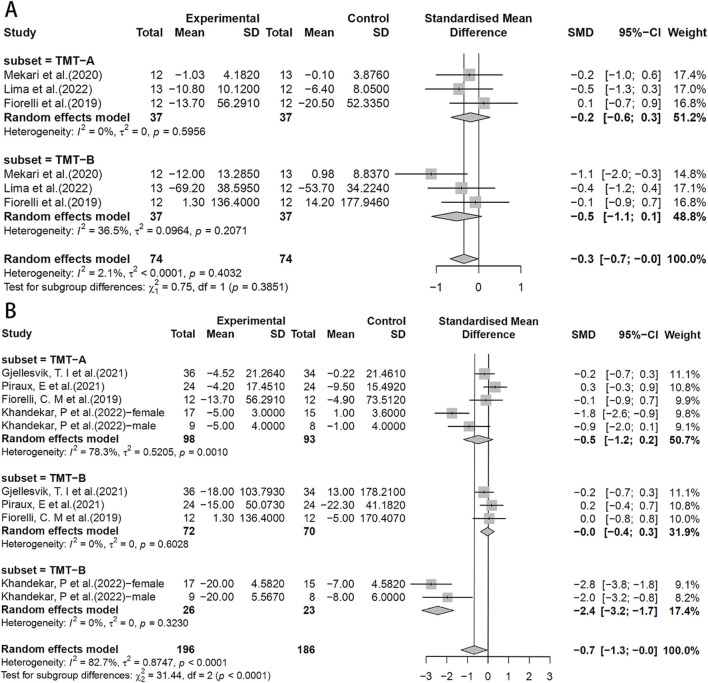
Forest plot for between-group effects of HIIT on the TMT test. **(A)** Comparison of HIIT and MICT in TMT test; **(B)** Comparison of HIIT and control in TMT test. TMT: Trail Making Test.

In the TMT test, the HIIT group exhibited a significant effect compared to the control group (SMD = −0.7, 95% CI: −1.3 to 0.0) (Figure 5B). Similarly, there were no significant differences between the HIIT group and the MICT group in the TMT test (Figure 5A).

**FIGURE 6:**
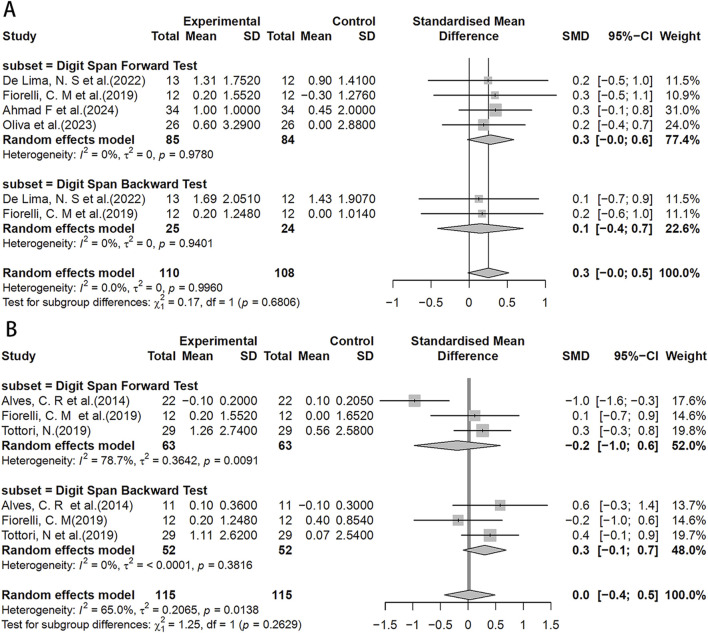
Forest plot for between-group effects of HIIT on the DST test. **(A)** Comparison of HIIT and MICT in DST test; **(B)** Comparison of HIIT and control in DST test. DST: Digit Span Test.

In the DST test, the HIIT group showed a potentially slightly better working memory span compared to the MICT group (SMD = 0.3, 95%CI: −0.0–0.5) (Figure 6A). However, no significant effects were found in the HIIT group when compared to the control group, although chance factors cannot be completely ruled out (Figure 6B).

**FIGURE 7:**
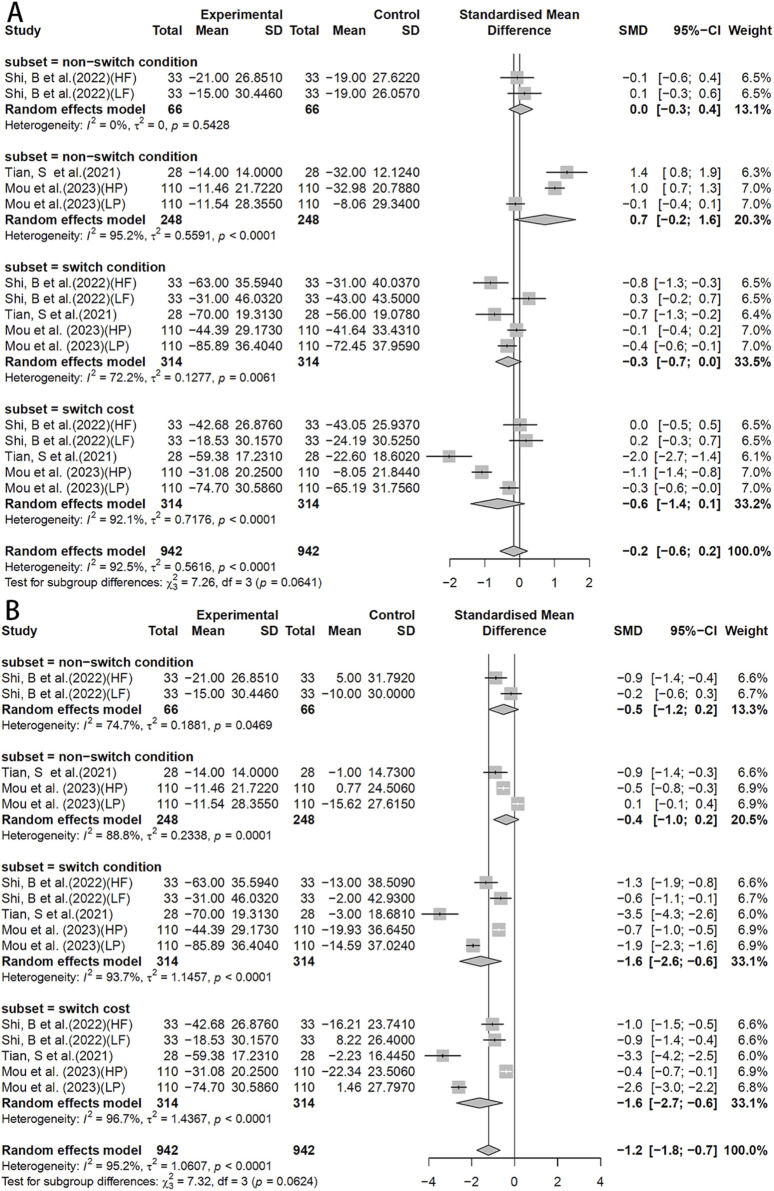
Forest plot for between-group effects of HIIT on the MOST test. **(A)** Comparison of HIIT and MICT in MOST test; **(B)** Comparison of HIIT and control in MOST test. MOST: The More-odd Shifting Task.

In the MOST test, no significant differences were observed between HIIT and MICT (Figure 7A), but a significant effect was observed in the HIIT group when compared to the control (SMD = −1.2, 95%CI: −1.8 to −0.7) (Figure 7B).

### 3.4 Sensitivity analysis

**FIGURE 8:**
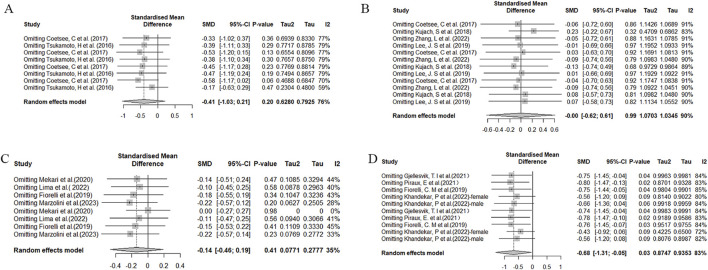
Sensitivity analysis of the effects of HIIT on Stroop and TMT tests. **(A)** Comparison of HIIT and MICT in Stroop test results; **(B)** Comparison of HIIT and control in Stroop test; **(C)** Comparison of HIIT and MICT in TMT test; **(D)** Comparison of HIIT and control in TMT test.

To assess the stability and reliability of the results obtained in this meta-analysis, a sensitivity analysis was conducted. Significantly heterogeneous findings were observed during functional assessments, specifically in the Stroop and TMT tests (Figure 8). This heterogeneity may be attributed to significant differences in factors such as age and training frequency among the study samples, as well as variations in experimental design methods. Specifically, in the Stroop test, differences were observed between the studies conducted by Tsukamoto, H (Tsukamoto et al., 2016) and Kujach, S (Kujach et al., 2018), which may have contributed to increased heterogeneity. In the case of the TMT test, the study conducted by Khandekar (Khandekar et al., 2023), P significantly differed from other studies, potentially contributing to increased heterogeneity.

**FIGURE 9:**
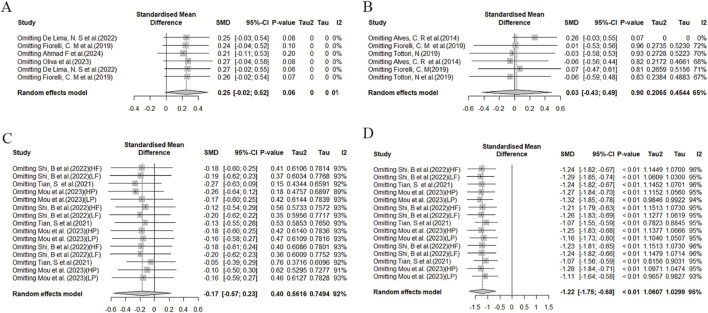
Sensitivity analysis of the effects of HIIT on MOST and DST tests. **(A)** Comparison of HIIT and MICT in DST test; **(B)** Comparison of HIIT and control in DST test; **(C)** Comparison of HIIT and MICT in MOST test; **(D)** Comparison of HIIT and control in MOST test.

Sensitivity analysis of the DST test (Figures 9A, B) reveals variability in effect sizes and observed heterogeneity across studies. In the MOST test (Figures 9C, D), we also observed high heterogeneity, which may stem from differences in experimental designs. For instance, Shi et al. (2022) employed a crossover design; Tian et al. (2021) utilized a within-subject repeated measures design.

Therefore, these differences and potential sources of heterogeneity should be considered when interpreting these findings. Due to limitations in sample size and study design, further research is needed to accurately understand and explain the observed heterogeneity.

## 4 Discussion

The present meta-analysis included 18 studies with a total of 827 patients. It compared the cognitive assessment results among the HIIT group, MICT group, and control group. We found that HIIT demonstrated better effects than MICT in the Stroop test (assessing attention control (Gignac et al., 2022) and inhibitory abilities (Chen et al., 2019; Daza González et al., 2021) and the DST test (assessing working memory (Sánchez-Cubillo et al., 2009; Llinàs-Reglà et al., 2017). Additionally, compared to the control group, HIIT showed significant effects in the TMT and MOST tests (evaluating cognitive flexibility (Tian et al., 2021; Shi et al., 2022; Zhang et al., 2022b; Xia et al., 2022), working memory (Zhang et al., 2022b), and inhibitory control (Zhang et al., 2022b). Overall, our study supports the effectiveness of HIIT in improving specific cognitive functions, particularly cognitive flexibility, working memory, task-switching abilities, attention control, and inhibitory control.

This meta-analysis aimed to systematically evaluate the effects of High-Intensity Interval Training (HIIT) on cognitive function, with a focus on executive function, which includes key domains such as working memory, inhibitory control, and cognitive flexibility. Our findings suggest that HIIT has a significant effect on certain aspects of cognitive function, particularly in executive control, as evidenced by the Stroop Test results, where HIIT significantly reduced neural response time compared to moderate-intensity continuous training (MICT). However, the effects of HIIT on other aspects, such as incongruent response time, accuracy, and interference response time, were not significant, indicating that the impact of HIIT on cognitive function is not uniform across all domains.

### 4.1 Overview of intervention characteristics

To provide clarity on the effectiveness of HIIT, it is important to consider the specific characteristics of the HIIT protocols used in the studies included in this meta-analysis. Most studies implemented HIIT sessions with varying duration, frequency, intensity, and types of intervals, which could contribute to the heterogeneity observed in our results. For instance, some studies employed walking or cycling as the primary mode of exercise, while others used running or uphill exercises. The frequency of HIIT sessions ranged from two to five times per week, with session durations ranging from 20 to 45 min. These variations in protocol design may explain some of the differences in the observed effects on cognitive function across studies.

### 4.2 Population characteristics

Our meta-analysis included studies involving a range of populations, including healthy adults, older adults, and individuals recovering from stroke. It is important to note that these populations may have different baseline cognitive function levels and responses to exercise. For example, studies involving older adults and stroke patients consistently demonstrated improvements in executive functions, such as inhibitory control and cognitive flexibility. These findings suggest that HIIT may be particularly beneficial for individuals with cognitive impairments or those at risk for cognitive decline.

### 4.3 Mechanisms underlying the cognitive effects of HIIT

Neuroplasticity: HIIT can significantly increase the levels of brain-derived neurotrophic factor (BDNF) (Jiménez-Maldonado et al., 2018; Hsu et al., 2021) which triggers a cascade of signaling pathways by activating its specific cell surface receptor, tropomyosin receptor kinase B (TrkB) (Casarotto et al., 2021). Which plays a crucial role in cell survival, anti-apoptosis, and neuronal growth and differentiation (Jin et al., 2022; Goyal et al., 2023).

Secondly, BDNF, through binding with the TrkB receptor, activates the MAPK/ERK signaling pathway (Li et al., 2021; Numakawa et al., 2018). In this process, a series of protein kinases are phosphorylated and activated. Additionally, BDNF, by activating the TrkB receptor, can trigger the PLCγ (Phospholipase C-gamma) pathway (Mohammadi et al., 2018). Activation and interaction of these signaling pathways are orderly and closely connected, collectively regulating various biological processes of neurons.

Cerebral hemodynamics: HIIT can enhance cardio-pulmonary function (Lavín-Pérez et al., 2021; Licker et al., 2017; Bönhof et al., 2022) by increasing cardiac output and cerebral blood flow (Lucas et al., 2015; Smith et al., 2021). The augmented blood circulation not only supplies neurons with additional energy sources like glucose and oxygen but also aids in clearing metabolic waste products such as beta-amyloid protein (associated with Alzheimer’s disease), thus mitigating their accumulation and neurotoxicity in the brain (Kisler et al., 2017; Qiang et al., 2017; Li et al., 2019). Moreover, the improved cerebral blood flow facilitates neuronal metabolism, synthesis, and release of neurotransmitters, thereby enhancing interneuronal information transmission (Kisler et al., 2017).

Neurochemical responses: HIIT can influence various neurotransmitters in the brain, such as serotonin, dopamine, and endorphins, which play crucial roles in emotional regulation (Anish, 2005; Zárate et al., 2002), attention, memory, and learning processes (Unger et al., 2020; Thiele and Bellgrove, 2018; Westbrook et al., 2021; Schoenfeld and Swanson, 2021). HIIT modulates the synthesis and release of these neurotransmitters through diverse pathways. Firstly, by inducing a stress response, HIIT triggers the release of stress hormones such as adrenaline and cortisol, thereby regulating the synthesis and release of serotonin and dopamine (de Oliveira Teles et al., 2022). Secondly, HIIT improves cardiovascular fitness by increasing cardiac output and cerebral blood flow, thereby facilitating neurotransmitter synthesis and release (Sultana et al., 2019)^.^ In addition, HIIT can impact neuronal excitability, making neurons more susceptible to activation, thereby stimulating the generation and release of a greater quantity of neurotransmitters.

Antioxidant and inflammatory responses: HIIT enhances the body’s antioxidant capacity by stimulating the production of antioxidant enzymes which can reduce damage to neurons caused by free radicals (Gholipour et al., 2022; Freitas et al., 2019; Souza et al., 2022). HIIT strengthens antioxidant capacity through multiple pathways. Firstly, during HIIT, muscle contractions and energy metabolism are activated, which induces intracellular stress responses. This stress response stimulates the production of antioxidant enzymes such as superoxide dismutase (SOD), catalase (CAT), and glutathione peroxidase (Gpx) (Fakhri et al., 2020; Azhdari et al., 2019).

### 4.4 Practical implications

While the current evidence is promising, the lack of standardization in HIIT protocols and population characteristics makes it difficult to draw firm conclusions about the most effective implementation of HIIT for cognitive improvement. Future research should aim to determine the optimal duration, frequency, and intensity of HIIT for improving executive function, especially in at-risk populations such as older adults and stroke survivors.

In the studies included in this meta-analysis, interventions varied in terms of duration, frequency, intensity, type of intervals, and exercise modality. The HIIT protocols generally involved sessions lasting between 4 and 12 weeks, with frequencies ranging from 2 to 5 sessions per week. Exercise intensity during HIIT was typically 80%–90% of maximum heart rate, with short bursts of high-intensity exercise followed by brief recovery periods. In contrast, MICT involved continuous moderate-intensity exercise, such as walking or cycling, at 50%–70% of maximum heart rate. These interventions were typically designed to be accessible to individuals with varying levels of fitness, with walking and cycling being the most common exercise types. Understanding the specific design of these interventions helps in determining the most appropriate and effective approach for different populations, highlighting the need for tailored recommendations for implementing HIIT and MICT in clinical and rehabilitation settings.

Overall, compared to MICT and a sedentary lifestyle, HIIT may enhance cerebral blood flow dynamics and neuroplasticity more effectively, leading to better improvements in cognitive performance. This does not imply, however, the benefits of MICT and other forms of exercise on health and cognitive function should be discounted. While HIIT has shown cognitive benefits, it is important to consider the value of moderate-intensity continuous training (MICT) as well. Both types of exercise offer unique advantages, with HIIT being time-efficient and MICT providing sustained benefits. Including both in an exercise regimen allows for a more tailored approach, ensuring that individuals at different fitness levels can benefit from both cognitive and physical health improvements. Additionally, given the high intensity of HIIT, individuals performing HIIT should adjust the intensity and frequency of exercise under medical guidance to avoid the risks of overtraining or injury.

Due to potential variations in the design and quality of the included primary studies, heterogeneity within certain subgroups may have influenced our results. Additionally, the small number of included studies and the inclusion of diverse populations (including healthy individuals, cancer patients, Parkinson’s disease patients, stroke patients, etc.,) may limit the generalizability of our findings. Furthermore, we did not extensively explore the effects of various forms and frequencies of HIIT on cognitive function, which limits our comprehensive understanding of the effects of HIIT. Lastly, since the majority of the study participants were young individuals, we did not fully consider the impact of age on the study outcomes.

## 5 Conclusion

Our meta-analysis demonstrates that HIIT significantly enhances specific cognitive functions, particularly cognitive flexibility, working memory, task-switching ability, attention control, and inhibitory control. This finding underscores the potential value of HIIT in improving specific cognitive tasks. Therefore, we recommend healthcare professionals consider incorporating HIIT into training programs when developing exercise plans. In the future, further high-quality research is needed to clarify the optimal patterns and frequencies of HIIT, as well as its applicability in different populations, thereby providing more comprehensive guidance for clinical and practical applications.

## Data Availability

The original contributions presented in the study are included in the article/supplementary material, further inquiries can be directed to the corresponding author.
